# The Pathology of Epilepsy

**Published:** 1882-01

**Authors:** 


### THE PATHOLOGY OF EPILEPSY.

L. Luciani concludes from a study of the literature of the subject as well as from his own investigations (published in the Rivista sperimentale de Frenatria e Medicina legalo, Anno iv., F. iv.) that the following propositions may be adduced concerning the pathogenesis of epilepsy:

*a.* Epilepsy of whatever form it may be, depends upon irritated condition of the cerebral cortex, together with a functional derangement of the psycho-motor centres.

*b.* This irritation may effect the whole cortical portion; it may limit itself to a single spot; or, spreading successively from point to point, it may invade the entire cortex.

*c.* When the irritation becomes sufficiently extended, there appears the ordinary form of epilepsy.

*d.* When the irritation is limited to a single psycho-motor centre, the spasm is also limited to the muscles supplied by it.

*e.* When the irritation extends from its original seat over other centres, the muscles will become affected in the same order.

*f.* In complete epilepsy, the centre affected can be determined with great certainty by reference to the muscles affected.

*g.* In the ordinary forms of epilepsy, this latter is of course not practicable.

It is here assumed that epilepsy is always of a cerebral origin. But when the cerebral cortex is very greatly destroyed, the epilepsy is no more severe. May not the epilepsy, then, be considered the morbid result of an increased functional activity of the psycho-motor centres? And when, after destruction of such a centre, well defined epilepsy is spontaneously developed, must it not be attributed essentially to this same centre? Luciani arrived at his conclusions after three experiments, in the third of which (performed upon a monkey), simple irritation of the cortical layer sufficed.

Here follows a controversy with Hitzig, to whom the author refers, he having claimed that the centre for an individual part is never completely destroyed. Corona holds the same view. As the arachnoidea fills the deep furrows between the convolutions in the dog, it is not an easy matter to destroy an entire convolution. In order, then to determine positively that you have destroyed an entire centre, the histological method may be employed (it gives the best results in the larger pyramidal ganglia), electricity being used, however, for the determination of individual centres. Luciani in this way was able to determine that by electrifying a single part of a motor zone, bilateral epileptic seizures may be produced; further, that after destruction of one side of this zone, there can be produced by irritation of the corresponding part of the opposite side a bi-lateral disturbance, or the ordinary epilepsy; and, lastly, that when the centre for only one hind leg remains, ordinary attacks can be developed by irritation of that centre. He rejects all other hypotheses and dwells at length upon the reflex action of the medulla oblongata already referred to. The co-operation of the latter is, however, only accessory and complimentary.

Finally, Luciani draws the following conclusions. The motor zone of the cortex represents the central organ for the epileptic convulsion. A morbid excitation of this zone, whether direct or indirect, of whatever origin and in whatever form exhibited, is the essential element in the epileptic process. A morbid stimulation of the medulla oblongata is probably an accessory of great importance, but not necessarily a part of the process.—*Cin. Lancet and Clinic.*

